# Influence of Human Platelet Lysate on Extracellular Matrix Deposition and Cellular Characteristics in Adipose-Derived Stem Cell Sheets

**DOI:** 10.3389/fcell.2020.558354

**Published:** 2020-10-22

**Authors:** Nai-Chen Cheng, Yuan-Kun Tu, Ning-Hsu Lee, Tai-Horng Young

**Affiliations:** ^1^Department of Surgery, National Taiwan University Hospital, National Taiwan University College of Medicine, Taipei, Taiwan; ^2^Department of Orthopedics, E-Da Hospital/I-Shou University, Kaohsiung, Taiwan; ^3^Institute of Biomedical Engineering, College of Medicine, College of Engineering, National Taiwan University, Taipei, Taiwan

**Keywords:** adipose-derived stem cell, cell sheet, extracellular matrix, human platelet lysate, fetal bovine serum, angiogenesis

## Abstract

Adipose-derived stem cell (ASC) is a valuable source of cell therapy. By stimulating extracellular matrix (ECM) secretion, ASC sheets can be fabricated with enhanced regenerative capabilities. In recent years, human platelet lysate (HPL) provides an attractive alternative to fetal bovine serum (FBS) for the *ex vivo* expansion of ASCs for clinical use. However, the effect of HPL on ASC sheet formation has not been previously determined. In this study, we compared ECM composition and cellular characteristics of ASC sheets cultured in growth medium supplemented with either FBS or HPL. HPL supplement significantly enhanced ASC proliferation without obvious change in the expression pattern of cell surface markers. We found that culturing ASCs with HPL rendered thicker cell sheets with significantly more ECM deposition, including collagen and fibronectin. Proteomic analysis of the FBS or HPL-cultured cell sheets showed diversity in ECM composition. HPL-cultured ASC sheets exhibited up-regulation of interleukin-6 and an anti-inflammatory cytokine, C1q/tumor necrosis factor-related protein-3. Conditioned medium of HPL-cultured ASC sheets significantly enhanced fibroblast migration and tube formation of endothelial cells *in vitro*, while it inhibited the migration of macrophages toward stimulated macrophages *in vitro*. TGF-β1-stimulated fibroblasts cultured in ASC sheet-conditioned medium showed down-regulation of *α-SMA* and *TGF-β1.* By adding an anti-hepatocyte growth factor (HGF) neutralizing antibody in conditioned medium, we indicated that an anti-fibrosis effect of HPL-cultured ASC sheets is partially mediated through the increased secretion of HGF. Moreover, chick embryo chorioallantoic membrane (CAM) assay showed comparable capillary density after applying either FBS or HPL-cultured ASC sheets, both of which were significantly higher than the control. In conclusion, robust ECM formation with altered ECM composition was noted in ASC sheets cultured in HPL-supplemented medium. Their immunomodulatory and pro-angiogenesis capabilities were largely maintained. Our findings paved the way to elucidate the potential of HPL-cultured ASC sheets for clinical application in tissue regeneration.

## Introduction

Cell therapy with mesenchymal stem cells (MSCs) has become a promising therapeutic approach for regenerative medicine. The abundance and easy accessibility of adipose-derived stem cell (ASC) have made it a promising candidate for MSC-based therapies. However, transplantation of dissociated ASCs is frequently associated with early cell death with limited therapeutic effects, and cell sheet engineering of ASCs holds promise in treating many diseases, including myocardial infarctions, skin wounds, hind limb ischemia, esophageal strictures, and oral ulcers (Sukho et al., 2018). We have successfully stimulated extracellular matrix (ECM) secretion of ASCs and fabricated cell sheets by applying a long-acting form of ascorbic acid, L-ascorbic acid 2-phosphate (A2-P), in the culture medium (Yu et al., 2014). With this approach, native ECM secreted by the cells was preserved, and ASCs could be harvested as a contiguous cell sheet with intact cell-to-cell connections. Enhanced expression of stemness markers, including Oct4, Sox2, and Nanog, was found in ASCs within the cell sheets. ASC sheets also possess the necessary paracrine factors to improve skin wound healing and decrease scar formation (Yu et al., 2018).

The aforementioned studies employed fetal bovine serum (FBS) for ASC culture during cell sheet fabrication. However, due to the risks of xeno-immunization against bovine antigens, transmission of zoonotic diseases, and potential limits of availability, it was necessary to find suitable human alternatives for the manufacture of ASC sheets to meet clinical therapeutic purposes (Mackensen et al., 2000; Horwitz et al., 2002; Schallmoser and Strunk, 2013; Barro et al., 2020). Human platelet lysate (HPL) contains a number of mitogenic growth factors, including fibroblast growth factor (FGF), endothelial growth factor (EGF), platelet-derived growth factors (PDGF), vascular endothelial growth factor (VEGF), and transforming growth factor (TGF; Fekete et al., 2012; Chen et al., 2018). The platelet lysates have demonstrated the advantages to replace FBS for culturing fibroblasts, endothelial cells or tumor cell lines (Burnouf et al., 2016; Schallmoser et al., 2020). A series of reports have shown that both allogeneic and autologous HPL are superior to FBS for stimulation of MSC proliferation (Schallmoser and Strunk, 2009; Naaijkens et al., 2012; Barro et al., 2019). Moreover, MSCs expanded in HPL-supplemented culture medium preserved their immune-privileged capabilities, including the suppression of T cell proliferation and activation (Flemming et al., 2011).

The highly stimulatory effect of HPL on cell proliferation enabled large-scale expansion of ASC for clinical use, yielding a great advantage when compared with the standard FBS culture method (Cowper et al., 2019; Gao et al., 2019). Besides enhancing the proliferative activity, HPL was also shown to promote ASC differentiation into osteoblasts and chondrocytes compared to FBS (Gao et al., 2019). HPL was suggested to alter the ASC behavior and function via multiple signaling pathways (Kakudo et al., 2019). A previous study showed that HPL can benefit cell behavior and ECM production, thus optimizing the conditions for cell sheet production in periodontal ligament stem cells (Tian et al., 2018). Since the application of HPL in fabricating ASC sheets had not been reported in the literature, it was therefore intriguing to explore the effect of replacing FBS with HPL on the microenvironment fabricated within ASC sheets. To elucidate the potential clinical application of HPL-cultured ASC sheets, we also examined their cellular function regarding wound healing, immunomodulation and angiogenesis.

## Materials and Methods

### Isolation and Culture of Human ASCs

Adipose-derived stem cells were isolated from the subcutaneous fat tissue of four non-smoking, non-diabetic female donors with an average age of 45 years (32–57 years) and an average body mass index of 24.6 (21.0–26.6) as described previously (Wu et al., 2020). The study protocol has been approved by the Research Ethics Committee of National Taiwan University Hospital, and the informed consent has been obtained from each donor of adipose tissue. This study was conducted in accordance to the institutional biosafety standards. Small pieces of subcutaneous fat tissue were finely minced and washed using phosphate-buffered saline (PBS; Omics Biotechnology, Taipei, Taiwan), followed by treatment with collagenase type I (Gibco, Carlsbad, CA, United States) for 60 min. FBS (Hyclone, Logan, UT, United States) was added to inhibit collagenase activity before the cell suspension was filtered and centrifuged. Pellets were suspended and plated with Dulbecco’s modified Eagle’s medium (DMEM)/F-12 (Hyclone) and supplemented with 10% FBS, 1% penicillin–streptomycin (Biological Industries, Kibbutz Beit Haemek, Israel), and 1 ng/mL fibroblast growth factor-2 (FGF2; R&D systems, Minneapolis, MN, United States). The cells were cultured in a 5% CO2 humidified atmosphere at 37 °C, and the medium was changed every 2–3 days. Upon reaching 90% confluence, the cells were detached using 0.05% trypsin-ethylenediaminetetraacetic acid (EDTA) (Biological Industries) and re-plated until the third or fourth passage for various experiments. The cells have been previously tested to exhibit differentiation capabilities toward osteogenic, chondrogenic and adipogenic lineages (Tsai et al., 2019). Isolated human ASCs were pooled to become a single population based on the comparable surface marker expression and differentiation potential presented in each ASC clone.

### Flow Cytometry Analysis and Population Doubling Assay

Commercially available HPL (UltraGRO^TM^, Helios BioScience, Atlanta, GA) was used for ASC culture supplement. ASCs were cultured in DMEM-high glucose (DMEM-HG; Hyclone) supplemented with 10% FBS, 5% HPL, 2% HPL or 1% HPL and subjected to flow cytometry analysis to determine the cell surface antigen expression. The cells were incubated with the following antibodies: human monoclonal antibodies against CD31 (BD Pharmingen, San Jose, CA, United States), CD44, CD34, CD73, CD90, and CD166 (all from BioLegend, San Diego, CA, United States). The samples were analyzed using a flow cytometer (FACSVerse; BD Biosciences, Franklin Lakes, NJ, United States) in which 10,000 cells were counted per sample. Positive cells were determined as the proportion of the population with higher fluorescence than 95% of the isotype control. Moreover, ASCs were seeded in 10 cm culture dishes and supplemented with 10% FBS, 5% HPL, 2% HPL, or 1% HPL. After 7 days, the cells were lifted, counted with Scepter 2.0 Cell Counter (Millipore, Billerica, MA, United States), and the population doubling time was calculated by the following formula:

$$\mathrm{Doubling}\,\mathrm{Time}\,\left(\text{h}\right)=168\times \mathrm{ln2}/\text{ln}\,\left(\text{Ne}/N_{0}\right)$$

*N*_0_ is the seeding cell number at the beginning of the incubation time (1.6 × 10^4^ cells per dish). Ne is the cell number at the end of the incubation time.

### Differentiation of Human ASCs

Adipose-derived stem cells were cultured in DEME-HG supplemented with 10% FBS or 5% HPL. When the cells reached 80% confluence, the medium was changed to respective induction medium. Adipogenic differentiation was induced by DEME-HG supplemented with 10% FBS or 5% HPL, 1% penicillin/streptomycin, 500 μM 3-isobutyl-1-methylxanthine, 1 μM dexamethasone, 10 μM insulin, and 400 μM indomethacin (all from Sigma). Osteogenic differentiation was induced by DMEM-HG supplemented with 10% FBS or 5%HPL, 1% penicillin/streptomycin, 10 nM dexamethasone, 50 μM A2-P, 10 nM 1α,25-dihydroxyvitamin D3, and 10 μM β-glycerophosphate (all from Sigma). ASCs were fixed in 4% paraformaldehyde and stained with Oil Red O (Sigma) for adipogenesis assay or Alizarin Red S (Sigma) for osteogenesis assay on day 14.

### Fabrication of ASC Sheets

To create cell sheets, ASCs were seeded at a density of 2.5 × 10^3^ cells/cm^2^. The culture medium consisted of DMEM-HG supplemented with 10% FBS or 5% HPL, 1% penicillin/streptomycin, and 250 μM A2-P. The culture medium was refreshed every 2∼3 days. The cell number within ASC sheets was estimated by measuring double-strand DNA (dsDNA) content of the ASCs. On day 7 and 14, DNA content was measured fluorometrically using the Quant-iT Picogreen dsDNA Reagent and Kits (Invitrogen, Carlsbad, CA, United States) according to the manufacturer’s protocol (excitation wavelength, 485 nm; emission wavelength, 535 nm). The cell number within each well was estimated using a calibration curve. Cell sheets were peeled off from the culture plates for further analysis on day 14.

### Microscopic Images and Histology

For the electron microscopic study, cell sheets fabricated in 10% FBS or 5% HPL were washed thoroughly and fixed in 4% paraformaldehyde in PBS for 20 min. The cell sheets were dehydrated by gradual change of concentrated ethanol, followed by lyophilization. The specimens were then sputter coated with platinum and examined using a scanning electron microscope (JSM-6700F, JOEL, Tokyo, Japan). A second harmonic generation microscopic system was also used to examine the ASC sheets as previously described (Wu et al., 2019). A wavelength-tunable Ti:Sapphire laser pumped by a diode-pumped solid-state laser (Spectra Physics, Mountain View, CA, United States) was used as the excitation source. The 880 nm output laser system was guided toward a modified upright microscope (BX51WI, Olympus, Tokyo, Japan). The excitation source was beam expanded and reflected toward the focusing objective by a primary dichroic beamsplitter (Semrock, Rochester, NY, United States). To optimize image quality without laser ablation, the laser power was set at about 70 mW on the tissue section. The non-linear autofluorescence signals were spectrally separated by bandpass filters of 434/17 nm and 510/84 nm (Semrock).

As for the histological analysis, FBS or HPL-cultured ASC sheets were fixed in 4% paraformaldehyde and then paraffin-embedded. Sections were cut perpendicular to the surface of the cell sheet with a thickness of 5 μm. Paraffin-embedded sections were de-parafinized, rehydrated, and stained with hematoxylin and eosin (H&E, Sigma) or Masson’s trichrome (Sigma). The thickness of cell sheets was determined using ImageJ. Three ASC sheets from each group were used, and five high-power fields in each section were randomly selected, followed by three measurements of sheet thickness per field.

### Ribonucleic Acid (RNA) Isolation and Quantitative Polymerase Chain Reaction (qPCR)

Total RNA was extracted from FBS or HPL-cultured monolayer ASCs and ASC sheets using a RNeasy Mini Kit (Qiagen, Valencia, CA, United States) according to the manufacturer’s protocols. After the RNA was isolated, complementary DNA (cDNA) was synthesized from the RNA using High-Capacity cDNA Reverse Transcription Kits (Applied Biosystems, Foster City, CA, United States). Real-time qPCR was performed with FastStart Universal SYBR Green Master (Roche, Indianapolis, IN, United States) using CFX Connect Real-Time PCR Detection System (Bio-Rad, Hercules, CA, United States). The expression level was analyzed and normalized to glyceraldehyde 3-phosphate dehydrogenase (GAPDH) for each cDNA sample. The relative quantity (RQ) of gene expression was calculated by relative quantification based on threshold cycle number. The sequences of the gene-specific primers are shown in Supplementary Table S1.

### Western Blot, ELISA, and Total Collagen Assay

The expression of collagen 1α1, fibronectin, laminin, and hepatocyte growth factor (HGF) was determined in FBS or HPL-cultured ASC sheets by western blot analysis. Briefly, ASC sheets were lysed in radioimmunoprecipitation assay buffer (Pierce Biotechnology, Rockford, IL, United States). After centrifugation, the protein content was determined in the supernatants by a bicinchoninic acid protein quantification kit (Pierce Biotechnology). Protein samples were added to sample buffer and boiled for 10 min. Proteins were separated by 7% sodium dodecyl sulfate polyacrylamide gel electrophoresis (SDS-PAGE) and blotted onto polyvinylidene difluoride membranes. Western blot was performed using anti-collagen 1α1 (R&D Systems, AF6220), anti-fibronectin (R&D Systems, MAB19182), anti-laminin (R&D Systems, AF4187), and anti-HGF (R&D Systems, AF-294-NA). Moreover, quantification of secreted HGF in the conditioned medium from FBS or HPL-cultured ASC sheets was performed using an ELISA kit for human HGF (Quantikine; R&D Systems). Data were expressed as the secreted factor per 10^5^ cells at the time of harvest.

Collagen content during the ASC sheet fabrication process was also assessed by Sircol collagen assay (Biocolor, Newtownabbey, United Kingdom). After 5 days of culture, cells in each well were detached by HyQtase (Hyclone) treatment and discarded, and the remaining ECM was digested with 5 mg/ml pepsin (Roche) overnight. Then 0.5 ml Sircol dye reagent was added into each well and agitated to form collagen-dye complex, followed by centrifugation and dye release by alkali treatment. The absorbance of the released dye was measured by a spectrometer (Infinite F200; Tecan, San Jose, CA, United States) at 570 nm.

### Proteomic Analysis of the ASC Sheet ECM Samples

Fetal bovine serum or HPL-cultured ASC sheets were decellularized with 0.5% Triton X-100 at 37°C for 10 min, and 0.3% ammonium hydroxide at 37°C for 5 min. The sheets were then treated with 240 U/ml of DNase-I for 1 h at 37°C as previously described (Lin et al., 2012). Decellularization was confirmed by 4’,6-diamidino-2-phenylindole (DAPI; Santa Cruz, Santa Cruz, CA, United States) staining under a fluorescence microscope. Protein samples were separated by SDS-PAGE, followed by in-gel digestion, then subjected to liquid chromatography-tandem mass spectrometry (LC-MS/MS). Proteins were then identified and quantified by proteomics softwares. The detailed experimental protocol is provided in the Supplementary Material.

### ASC Sheet-Conditioned Medium for Fibroblast Culture

Conditioned media were harvested from ASC sheets cultured in 10% FBS or 5% HPL after culturing for 24 h. Terminally differentiated human fibroblasts (Hs68) were seeded into a 24-well culture plate with a small rectangular culture inserts (Ibidi, Gräfelfing, Germany) placed at the center of each well. The seeding density was 2 × 10^4^ cells/well, and the cells were incubated in DMEM-HG supplemented with 10% FBS and 1% penicillin/streptomycin for 4 h to allow attachment. Then the inserts were removed, creating a rectangular cell-free zone in each well. After washing twice with PBS, the medium was changed to the collected conditioned medium of FBS or HPL-cultured ASC sheets. Serum-free DEME-HG was employed as a control group. Time-lapse microscopic images were taken with a time interval of 60 min for 12 h using an inverted microscope, and *in vitro* migration of fibroblasts into the cell-free zone was quantified using ImageJ.

The effect of ASC sheet-conditioned medium on Hs68 cell proliferation was also examined. Hs68 fibroblasts were seeded at a density of 2 × 10^4^ cells/well in 24-well culture plates. After cell attachment, the medium was replaced with the conditioned medium of FBS or HPL-cultured ASC sheets. On days 1, 4, 7, alamar blue solution (AbD Serotec, Kidlington, United Kingdom) was directly added into the culture wells, and the plate was further incubated at 37°C for 24 h. The fluorescence signals are measured at an excitation wavelength at 560 nm and an emission wavelength at 590 nm by a spectrometer. Moreover, Hs68 cells were stimulated with 20 ng/mL TGF-β1 for 24 h to test the anti-fibrosis effect of ASC sheet-conditioned media. Subsequently, the medium was replaced by the conditioned medium from FBS or HPL-cultured ASC sheets supplemented with 20 ng/mL TGF-β1. In two groups, anti-human HGF neutralizing antibody (5 μg/mL; Sigma, H0652) was also added in the ASC sheet-conditioned media. After 40 h, Hs68 cells were harvested for analysis of *TGF-β1* and α-smooth muscle actin (*α-SMA*) gene expression.

### ASC Sheet-Conditioned Medium for Macrophage Chemotaxis Assay

The influence of conditioned medium derived from monolayer ASCs and ASC sheets on the chemotaxis of mouse macrophage J774A1 cells (Bioresource Collection and Research Center, Hsinchu, Taiwan) was determined using transwell inserts with 8-μm pores (Corning). J774A1 cells which had been stimulated by lipopolysaccharide (LPS; 0.1 μg/mL) for 4 h were seeded in the lower wells at a density of 3 × 10^5^ cells/well. The upper wells were seeded with unstimulated macrophages at a density of 1 × 10^5^ cells/well, and the medium was replaced with the conditioned media of FBS or HPL-cultured monolayer ASCs or ASC sheets. After 24 h of culture in conditioned medium, macrophage migration through the transwell membrane was assessed through crystal violet (Sigma) staining of the lower surface of the membrane. The stained cells in the acquired microscopic images were quantified using ImageJ.

### ASC Sheet-Conditioned Medium for *in vitro* Tube Formation Assay

Human umbilical vein endothelial cells (HUVECs) were seeded on μ-slide (Ibidi) at a density of 8,000 cells/well, which were previously coated with Matrigel (Corning, Lowell, MA, United States). HUVECs were treated with the conditioned medium of FBS or HPL-cultured ASC sheets. All conditioned medium was mixed with equal volume of endothelial growth medium 2 (EGM2; PromoCell, Heidelberg, Germany) and applied for HUVEC culture. A basal medium mixed by equal volume of DMEM-HG and endothelial basal medium (EBM, PromoCell) served as a negative control, while EGM2 was used as a positive control. Formation of tube-like structures was visualized by a phase-contrast microscope at 6 h, and the images were analyzed using ImageJ.

### Angiogenesis Assay in Chick Embryo

The chick embryo chorioallantoic membrane (CAM) model is a well-established *in ovo* assay for studying angiogenesis (Cheng et al., 2017). Briefly, fertilized chicken eggs were incubated at 37°C with 70% humidity. On day 3, a circular window was made on the air chamber, and the embryo viability was evaluated. On day 7, FBS or HPL-cultured ASC sheets attached to polyester membranes (Corning) as carriers were placed onto the CAM through the open window. The opening window in the shell was then sealed with Tegaderm (3M) to prevent dehydration and contamination, and the eggs were returned to the incubator at 37°C for another 3 days. On day 10, the embryos were infused with 4% paraformaldehyde and placed at −80°C overnight. ASC sheets and adjacent CAM tissues were removed and transferred to 6-well plates containing 4% paraformaldehyde. The CAM specimens were photographed, and the blood vessels were quantified by measuring the capillary area and length, as well as counting the number of capillary branch points and nodes using ImageJ.

### Statistical Analysis

All investigations were confirmed by at least three independent experiments. All measurements are presented as the means ± standard deviation. Statistical significance was evaluated using an independent-sample Student’s t-test or one-way ANOVA. Tukey’s post hoc test was used when the group of interest was compared to all other groups in the experiment. All statistical analyses were performed using GraphPad Prism 7 (La Jolla, CA, United States), and statistically significant values were defined as *p* < 0.05.

## Results

### Characterization of FBS and HPL-Cultured ASCs

**FIGURE 1:**
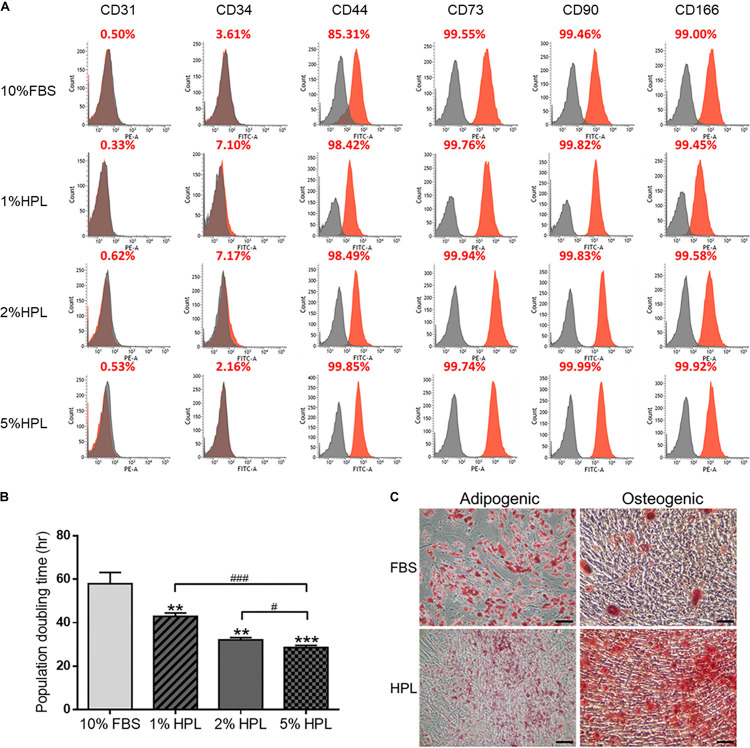
Phenotypic characteristics of adipose-derived stem cells (ASCs) cultured in medium supplemented with fetal bovine serum (FBS) or different concentrations of human platelet lysate (HPL). **(A)** Representative flow cytometry results of ASC surface antigens shown as the proportion relative to a positive control. **(B)** Population doubling time of ASCs cultured with FBS or different concentrations of HPL (*n* = 3; *p < 0.05; **p < 0.01; ***p < 0.005 from 10% HBS, ^#^*p* < 0.05; ^##^*p* < 0.01; ^###^*p* < 0.005 between the indicated groups). **(C)** Microscopic images of FBS or HPL-cultured ASCs cultured in adipogenic and osteogenic induction medium for 14 days. Adipocytes and osteocytes were stained by Oil Red O and Alizarin red, respectively (scale bar = 100 μm).

Human ASCs cultured with 10% FBS, 5% HPL, 2% HPL or 1% HPL were harvested for flow cytometry analysis. The surface epitopes of HPL-treated ASCs were similar to those of ASCs undergoing FBS treatment. These ASCs were negative for hematopoietic markers CD31 and CD34, and they were positive for MSC-related markers CD44, CD73, CD90, and CD166 (Figure 1A). Population doubling time estimated by cell number calculation demonstrated higher ASC proliferative activity under HPL culture condition (1% HPL: 43.0 ± 0.9 h, 2% HPL: 32.1 ± 0.6 h, 5% HPL: 28.6 ± 0.5 h vs. 10% FBS: 58.0 ± 3.0 h, *p* < 0.01, respectively; Figure 1B). Since significant statistical difference was found among the population doubling time of the 1, 2, and 5% HPL groups, 5% HPL was chosen to be used in the subsequent experiments. Moreover, 10% FBS or 5% HPL-treated ASCs maintained their adipogenic and osteogenic differentiation capabilities after application of appropriate induction media, as demonstrated by histology staining specific for oil and calcium, respectively (Figure 1C).

### HPL Enhanced Cell Sheet Formation

**FIGURE 2:**
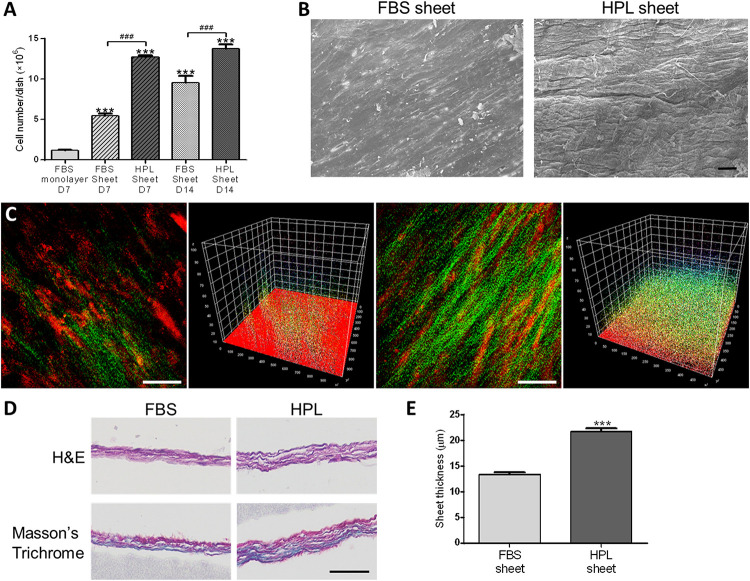
Cell sheet formation of fetal bovine serum (FBS) or human platelet lysate (HPL)-cultured adipose-derived stem cells (ASCs). **(A)** Representative scanning electron microscopic images of FBS or HPL-cultured ASC sheets (scale bar = 50 μm). **(B)** Cell number estimated by double-strand DNA (dsDNA) quantification assay (*n* = 3). **(C)** Second harmonic generation images of FBS or HPL-cultured ASC sheet after 7 days of culturing (scale bar = 50 μm). **(D)** Hematoxylin and eosin (H&E) and Masson’s Trichrome staining of cross section after 14 days of FBS or HPL-cultured ASC sheets (scale bar = 50 μm). **(E)** Thickness of the ASC sheets measured in histological images (*n* = 6; ***p < 0.005 from the control, ###*p* < 0.005 between the indicated groups).

Cell number within FBS or HPL-cultured ASC sheets were estimated by dsDNA assay, revealing significantly more cells in the HPL group relative to the FBS group on either day 7 or 14 of culture (Figure 2A). Under electron microscope, HPL-cultured cell sheets appeared to exhibit more matrix, and it was difficult to distinguish the cellular boundary on the sheet surface (Figure 2B). Comparing to FBS-cultured ASC sheets, abundant collagen deposition was noted in the HPL-cultured ASC sheets under second harmonic imaging microscopy (Figure 2C). Cell sheets were also peeled off from the tissue culture plates and subjected to histological examination of their cross sections. H&E staining showed 2 or 3 layers of ASCs within the FBS-cultured cell sheets, while 3 to 4 layers of ASCs could be observed in the HPL-cultured cell sheets. Masson’s trichrome staining demonstrated abundant ECM formation within the interstitial voids of the HPL-cultured ASC sheets (Figure 2D). Moreover, quantitative measurement of the thickness of the cell sheets revealed significantly thicker sheet formation in the HPL group (13.4 ± 0.5 μm vs. 21.8 ± 0.6 μm, *p* < 0.005; Figure 2E).

**FIGURE 3:**
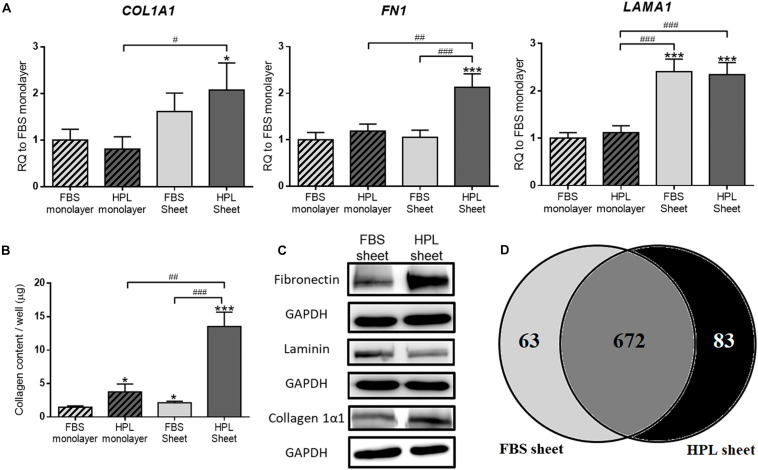
Extracellular matrix (ECM) deposition in fetal bovine serum (FBS) or human platelet lysate (HPL)-cultured adipose-derived stem cell (ASC) sheets. **(A)** Gene expression of collagen 1α1 (*COL1A1*), fibronectin (*FN1*), and laminin (*LAMA1*) in ASCs under different culture condition (*n* = 3). **(B)** Sircol assay of ASCs cultured under different culture condition (*n* = 3). **(C)** Western blot analysis of the expression of the ECM proteins in FBS or HPL-cultured ASC sheets. **(D)** Venn diagram of the number of proteins detected on LC/MS in the ECM of ASC sheets cultured with FBS and HPL, respectively (*p < 0.05; **p < 0.01; ***p < 0.005 from control, ^#^*p* < 0.05; ^##^*p* < 0.01; ^###^*p* < 0.005 between the indicated groups).

Real-time qPCR analysis revealed 2.1 ± 0.3-fold up-regulated collagen 1α1 (*COL1A1*), 2.1 ± 0.2-fold up-regulated fibronectin (*FN1*) and 2.3 ± 0.2-fold up-regulated laminin (*LAMA1*) genes in HPL-cultured ASC sheets relative to FBS-cultured monolayer ASCs, and significantly more transcripts of these genes were also noted when compared to the HPL-cultured monolayer ASCs (Figure 3A). Relative to FBS-cultured ASC sheets, HPL-treated ASC sheets exhibited significant higher collagen contents as determined by Sircol collagen assay (*p* < 0.005; Figure 3B). Western blot results also revealed enhanced collagen 1α1 and fibronectin expression in HPL-cultured ASC sheets (Figure 3C). Through proteomic analysis, we further identified 63 unique proteins in FBS-cultured ASC sheets and 83 unique proteins in HPL-treated ASC sheets (Figure 3D and Supplementary Table S2).

### Conditioned Medium Enhanced Fibroblast Migration and Proliferation

**FIGURE 4:**
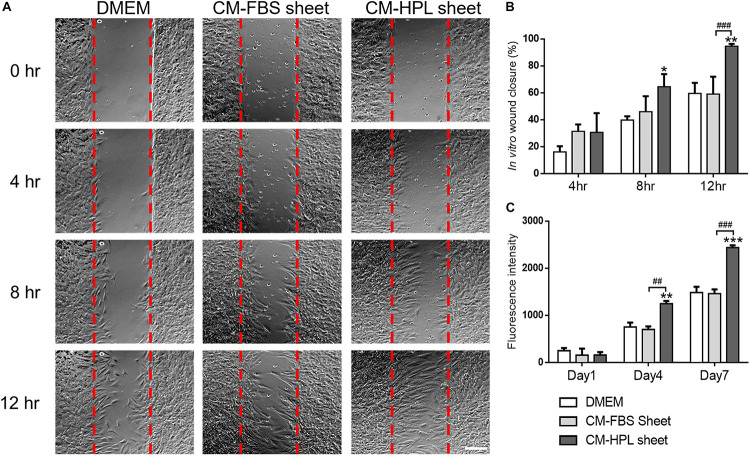
Effect of conditioned medium (CM) collected from fetal bovine serum (FBS) or human platelet lysate (HPL)-cultured adipose-derived stem cell (ASC) sheets on migration and proliferation of HS68 fibroblasts. **(A)** Representative microscopic images of *in vitro* cell migration assay. Serum free Dulbecco’s modified Eagle’s medium (DMEM) was employed as the control group (scale bar = 200 μm). Red dotted lines represent the initial borders of the cell-free zone. **(B)** Quantification of the wound closure percentage of Hs68 fibroblasts. **(C)** Alamar blue assay for estimating Hs68 cell proliferation (*n* = 3; *p < 0.05; **p < 0.01; ***p < 0.005 from DMEM control, ^#^*p* < 0.05; ^##^*p* < 0.01; ^###^*p* < 0.005 between the indicated groups).

Incubated in conditioned medium of FBS or HPL-cultured ASC sheets, Hs68 fibroblasts were subjected to *in vitro* cell migration assay (Figure 4A). The migrated fibroblasts covered a significantly larger portion of wound area in the group of HPL-cultured ASC sheet (FBS sheet: 59.0 ± 13.0% vs. HPL sheet: 94.52 ± 1.5% at 12 h, *p* < 0.005; Figure 4B). Moreover, alamar blue assay revealed significantly higher Hs68 cell proliferation when cultured with conditioned medium from HPL-sheets at days 4 and 7 (*p* < 0.01 and *p* < 0.005 relative to the FBS group at each day; Figure 4C).

### Immunomodulatory Gene Expression in HPL-Cultured ASC Sheets

**FIGURE 5:**
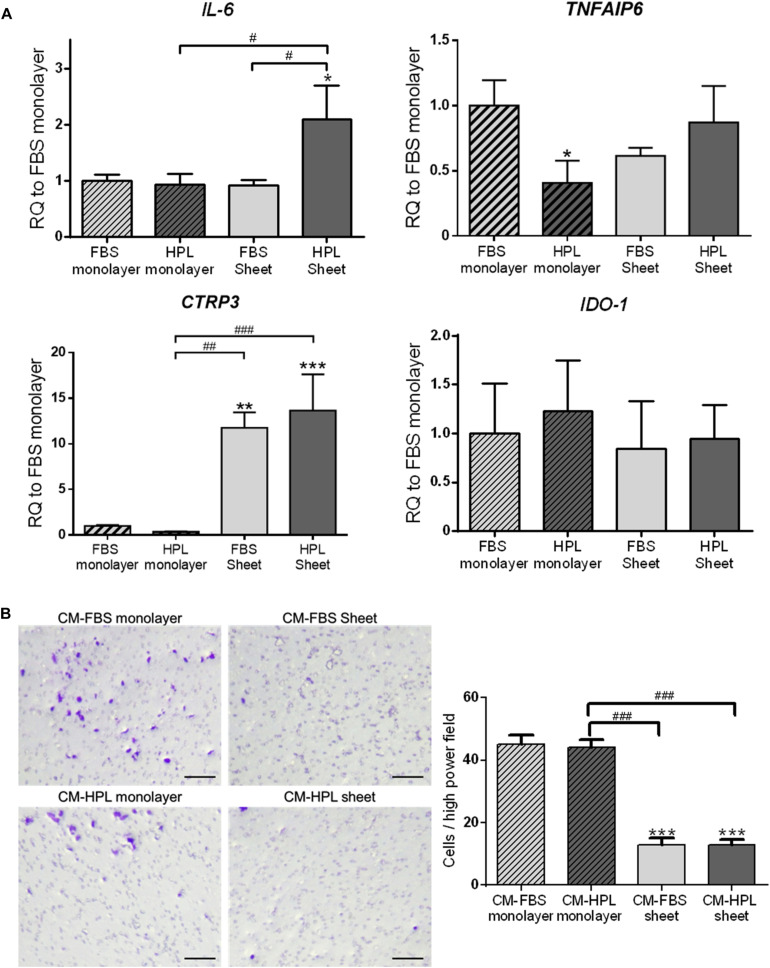
Expression of immunomodulatory factors in fetal bovine serum (FBS) or human platelet lysate (HPL)-cultured ASC sheets and their influence on macrophage migration. **(A)** Real-time quantitative polymerase chain reaction (qPCR) measurements for immunomodulatory genes in monolayer and ASC sheets during *in vitro* culture. **(B)** Migration of macrophages toward lipopolysaccharide (LPS)-stimulated mouse macrophages was significantly inhibited by conditioned medium (CM) of FBS or HPL-cultured ASC sheets (*n* = 3; *p < 0.05; **p < 0.01; ***p < 0.005 from FBS monolayer group, ^#^*p* < 0.05; ^##^*p* < 0.01; ^###^*p* < 0.005 between the indicated groups).

In order to fully understand the immunomodulatory capabilities associated with FBS or HPL-cultured ASC sheets, we examined the expression of immunomodulation-related genes, including interleukin-6 (*IL6*), tumor necrosis factor-α-induced protein 6 (TSG-6; *TNFAIP6*), C1q/tumor necrosis factor-related protein-3 (*CTRP3*), and indoleamine 2,3-dioxygenase (*IDO-1*). Relative to the FBS monolayer culture condition, FBS-cultured ASC sheets showed significant up-regulation of *CTRP3* (11.8 ± 1.7-fold, *p* < 0.005), while HPL-cultured ASC sheets exhibited significant up-regulation of both *IL-6 and CTRP3* (2.1 ± 0.6-fold, *p* < 0.05 and 13.7 ± 4.0-fold, *p* < 0.005, respectively; Figure 5A).

Moreover, we collected conditioned medium of FBS or HPL-cultured monolayer ASCs or ASC sheets and applied in a transwell experimental setting of macrophage culture *in vitro*. The chemotaxis of macrophages toward LPS-stimulated macrophages was examined, and the number of migrated cells on the undersurface of the transwell membrane was estimated. Relative to the groups using monolayer ASC-conditioned media, the migrated cell number was significantly lower in the presence of ASC sheet-conditioned media relative to the control (*p* < 0.005 respectively). Among them, the FBS and HPL-cultured ASC sheet-conditioned medium groups exhibited no significant difference in the number of migrated cells (Figure 5B).

### Expression of Angiogenic Growth Factors in HPL-Cultured ASC Sheets

**FIGURE 6:**
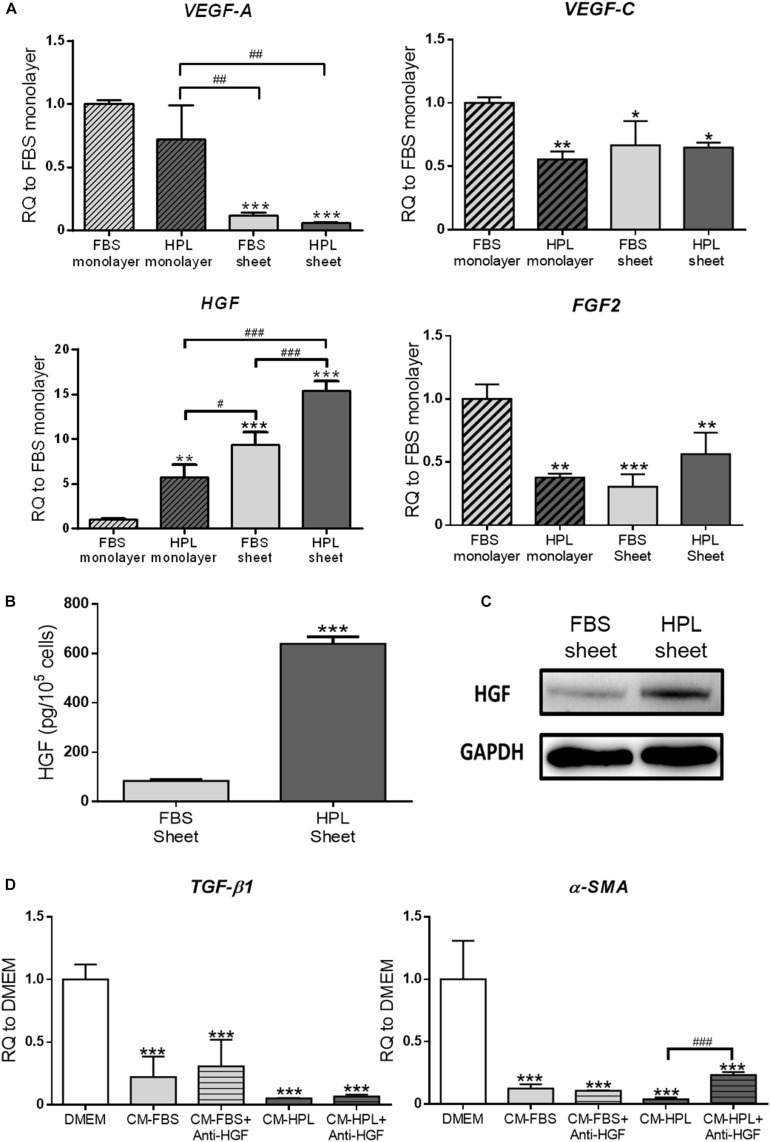
Enhanced secretion of hepatocyte growth factor (HGF) from adipose-derived stem cell (ASC) sheets cultured in human platelet lysate (HPL). **(A)** Gene expression of several angiogenic growth factors in ASCs under different culture condition (*n* = 3; *p < 0.05; **p < 0.01; ***p < 0.005 from fetal bovine serum (FBS) monolayer group, ^#^*p* < 0.05; ^##^*p* < 0.01; ^###^*p* < 0.005 between the indicated groups). **(B)** Release of HGF from FBS or HPL-cultured ASC sheets as determined by ELISA (*n* = 3; *** *p* < 0.005 from FBS sheet). **(C)** Western blot analysis of HGF expression in FBS or HPL-cultured ASC sheets. **(D)** Gene expression of *TGF-β1* and *α-SMA* in TGF-β1-stimulated Hs68 fibroblasts treated by different conditioned medium (CM) with or without anti-HGF neutralizing antibody (*n* = 3; ***p < 0.005 from Dulbecco’s modified Eagle’s medium (DMEM) group; ^###^*p* < 0.005 between the indicated groups).

The relative mRNA expression of several angiogenic growth factors, including VEGF-A, VEGF-C, HGF, and FGF2, was analyzed by real-time qPCR. Relative to the FBS monolayer culture group, down-regulation of *VEGF-C* and *FGF2* was noted in HPL treatment or sheet formation groups. *VEGF-A* transcript number was significantly lower in the FBS or HPL-cultured ASC sheets, while up-regulation of *HGF* was noted in both groups. Compared to FBS-cultured ASC sheets, the HPL counterpart exhibited a significantly higher expression level of *HGF* (9.4 ± 1.4-fold vs. 15.4 ± 1.1-fold, *p* < 0.005; Figure 6A). ELISA analysis of HGF levels in human ASC sheet-conditioned media confirmed higher content of HGF released from HPL-cultured ASC sheets (FBS sheet: 84.2 ± 13.5 pg/10^5^ cells vs. HPL sheet: 660.0 ± 47.1 pg/10^5^ cells, *p* < 0.005; Figure 6B). Western blot analysis also showed more HGF expression in HPL-cultured ASC sheets compared to the FBS-cultured sheets (Figure 6C). Significant down-regulation of *α-SMA* and *TGF-β1* was noted in *TGF-β1*-stimulated Hs68 fibroblasts treated by conditioned medium of either FBS or HPL-cultured ASC sheets. Moreover, the down-regulation of *α-SMA* was partially reversed in Hs68 fibroblasts after supplementing anti-HGF neutralizing antibody in the conditioned medium of HPL-cultured ASC sheets (Figure 6D).

### Tube Formation Assay of Endothelial Cells

**FIGURE 7:**
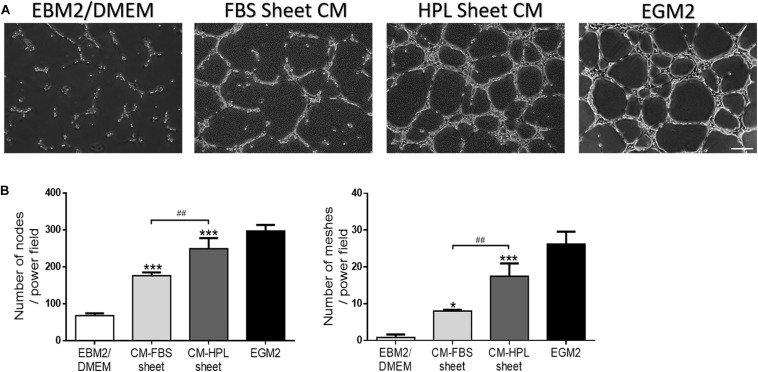
Effect of conditioned medium (CM) collected from fetal bovine serum (FBS) or human platelet lysate (HPL)-cultured adipose-derived stem cell (ASC) sheets on the *in vitro* tube formation of Human umbilical vein endothelial cells (HUVECs). **(A)** Representative microscopic images of endothelial cells after incubation with conditioned medium. HUVECs cultured in endothelial growth medium 2 (EGM2) served as a positive control, and those in EBM2/DMEM was a negative control (scale bar = 200 μm). **(B)** Number of branching nodes and meshes per power field were compared among different groups. Values are presented as means ± SD (*n* = 3; *p < 0.05; **p < 0.01; ***p < 0.005 from EBM2/DMEM control, ^#^*p* < 0.05; ^##^*p* < 0.01; ^###^*p* < 0.005 between the indicated groups).

Conditioned media harvested from FBS or HPL-cultured ASC sheets were used for HUVEC culture, and the endothelial cells began to form a vascular network structure within 4 h (Figure 7A). The *in vitro* tube formation of HUVECs was quantified by counting the total number of branching nodes and meshes per power field. Relative to the group receiving conditioned medium of FBS-cultured ASC sheets, the HPL group exhibited significantly more tube-like structures (176.2 ± 6.9 vs. 249.3 ± 23.3 for nodes; 8.0 ± 0.3 vs. 17.4 ± 2.9 for meshes, *p* < 0.01, respectively; Figure 7B).

### ASC Sheets Enhanced Angiogenesis in CAM Assay

**FIGURE 8:**
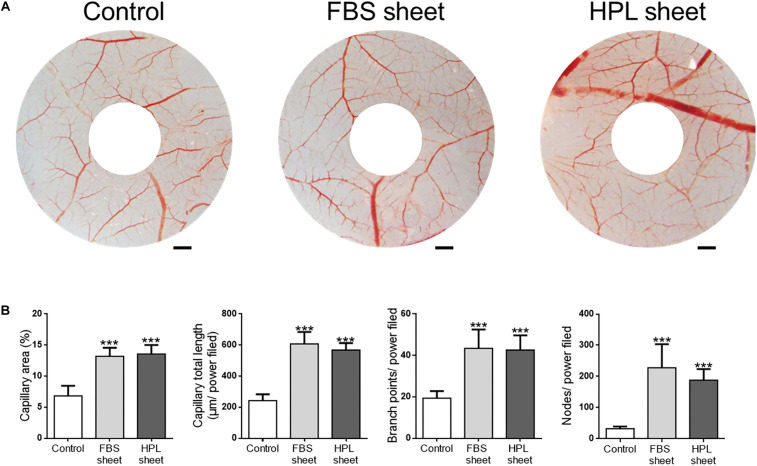
Chick embryo chorioallantoic membrane (CAM) assay. **(A)** Photographs of CAMs after treatment with fetal bovine serum (FBS) or human platelet lysate (HPL)-cultured adipose-derived stem cell (ASC) sheets, which were loaded on the CAMs of day 7 chick embryos. After 72 h of incubation, CAMs were excised and photographed (scale bar = 2 mm). **(B)** Blood vessel formation on CAMs was quantified by measuring the area covered by capillaries and the total capillary length. The number of capillary branch points and nodes per power field were also counted (image analysis performed on 10 random fields per sample, *n* = 3; ***p < 0.005 from control).

We examined the capillary formation on CAM to investigate the angiogenesis potential of FBS or HPL-cultured ASC sheets (Figure 8A). After implantation of FBS or HPL-cultured ASC sheets on CAMs, the capillary area proportion around the sheets was estimated to be significantly higher relative to the control (FBS sheet: 13.2 ± 0.6%, HPL sheet: 13.6 ± 0.7% vs. control: 6.8 ± 1.6%, *p* < 0.005, respectively; Figure 8B). Total capillary length around the sheets on CAMs was also estimated to be significantly longer relative to the control (FBS sheet: 607 ± 77 μm per power field, HPL sheet: 568 ± 45 μm per power field vs. control: 242 ± 41 μm per power field; *p* < 0.005, respectively). Similarly, placing ASC sheets on CAMs also yielded significantly more capillary branch points or nodes per power field (*p* < 0.005 respectively; Figure 8B).

## Discussion

By preserving the cellular junctions and endogenous ECM, cell sheets recreate cellular microenvironments *in vitro*, mimicking the inherent chemical, mechanical, and biological properties of cellular niche. Therefore, cell sheet fabrication is deemed beneficial for ASC-based regenerative therapies (Sukho et al., 2018). In our previous studies, transferable ASC sheets can be generated by employing A2-P in the culture medium, and they exhibited enhanced biological function to promote wound healing and decrease scar formation. We also revealed the important role of endogenous ECM in the enhanced stemness of ASCs within cell sheets (Yu et al., 2014, 2018). Given the application of ASC sheets in clinical settings, they would ideally be generated without using xenogeneic supplements such as FBS. HPL has been shown to surpass xenogeneic FBS in terms of bio-safety, while maintaining the potential of ASCs for proliferation, differentiation and immunomodulation (Cowper et al., 2019; Kakudo et al., 2019). Therefore, adopting HPL is a viable alternative for ASC sheet fabrication, which can facilitate the translation of this technology into clinical treatments. By comparing the ASC sheets cultured with FBS or HPL, this study revealed prominent differences in the abundance of ECM and the composition of the microenvironment deposited *in vitro*.

Human platelet lysate prepared from human platelets has been increasingly suggested to replace FBS in clinically oriented MSC expansion applications, allowing efficient cell propagation under animal serum-free conditions for clinical use (Cowper et al., 2019; Pasztorek et al., 2019; Schallmoser et al., 2020). Although autologous blood is a reliable resource for producing HPL, requesting each patient to donate his/her blood during the long-term *in vitro* cell expansion process is certainly not cost effective. Therefore, large scale and standardized manufacturing of allogeneic HPL products from platelets of human blood donors for cell culture is a more practical option (Glovinski et al., 2017), and the potential risk of viral contamination should be judiciously evaluated during the manufacturing process (Burnouf et al., 2019). The expression of cell surface markers and adipogenic/osteogenic differentiation capabilities were maintained after replacing FBS with HPL for ASC culture. In a collaborative work, we have previously shown that HPL promoted human ASC differentiation into osteoblasts and chondrocytes compared to FBS (Gao et al., 2019). With 5% HPL employed in this study, the proliferative activity of ASCs was significantly increased relative to 10% FBS. The finding was in line with a previous study showing that 5% HPL was the optimal concentration for culturing human ASCs (Kakudo et al., 2019). Consequently, significantly more cells were found within the ASC sheets fabricated with HPL relative to the FBS-cultured cell sheets.

Histology of the cell sheet cross sections revealed significantly thicker HPL-cultured cell sheets relative to the FBS-cultured counterparts, comprising of several layers of ASCs with abundant ECM formation within the interstitial voids of the cells. Abundant deposition of ECM molecules, including collagen and fibronectin, was also found in the HPL-cultured ASC sheets. We previously demonstrated that A2-P-induced ECM synthesis was crucial for stemness enhancement of ASCs within cell sheets, indicating the importance of the microenvironment formulated by the ASC-secreted ECM (Yu et al., 2014). ECM signals are transduced into cells by cell surface receptors, thereby affecting various cellular functions. Stem cell fate determination by the surrounding ECM may be mediated through physical cell support and control of cell shape and geometry as a function of surface topography, substrate stiffness, and biochemical signaling (Engler et al., 2006). Stem cell-derived ECM was found to display a specific and unique matrisome signature, leading to differential survival and gene-expression profiles among the cell types (Ragelle et al., 2017). In this study, in addition to the different expression quantities of several important ECM proteins, mass spectrometry-based proteomic analysis further revealed that some proteins were found exclusively in the FBS or HPL-cultured ASC sheets. By allowing interactions of cells with the surrounding ECM to adapt to their native morphology, the cell-derived ECMs can influence the resident cells in a different manner (Cheng et al., 2012). Therefore, certain differences are expected between the cellular phenotype and biological function of ASCs in different microenvironment of FBS or HPL-cultured cell sheets.

We previously demonstrated that FBS-cultured ASC sheets enhanced skin wound healing, and the neodermis possessed a highly organized collagen structure (Yu et al., 2018). In this study, HPL-cultured ASC sheets were found to be more effective in stimulating the migration and proliferation of fibroblasts *in vitro*, indicating their potential application in wound healing. Moreover, an anti-scarring effect of the ASC sheets-conditioned medium was evidenced by the down-regulation of *TGF-β1* and *α-SMA* in TGF-β1-stimulated fibroblasts. After an anti-human HGF neutralizing antibody was added in the conditioned medium of HPL-cultured ASC sheets, partial reverse of the *α-SMA* down-regulation was observed in TGF-β1-stimulated fibroblasts. The finding indicates that HPL-cultured ASC sheets inhibit the differentiation of fibroblasts toward α-SMA-expressing myofibroblasts partially through the increased secretion of HGF. Similarly, Ma et al. recently demonstrated that ASC-secreted HGF effectively inhibits fibrosis-related factors and regulates ECM remodeling in hypertrophic scar fibroblasts (Ma et al., 2020).

The immunomodulatory function of ASCs has been recognized to play important roles for their therapeutic activity (Prockop and Oh, 2012). In an inflammatory microenvironment, ASCs can profoundly affect the function of various effector cells involved in the adaptive and innate immune reaction, including T-cells, B-cells, natural killer (NK) cells, monocytes, macrophages, dendritic cells, neutrophils, and mast cells (Prockop and Oh, 2012; Hsu et al., 2013). The specific molecular mechanisms involved in the immune regulatory properties of ASCs are associated with both cell contact-dependent mechanisms and soluble inducible factors. ASCs release an array of cytokines that exert potent immunomodulatory effects capable of regulating multiple signaling pathways and cell types that contribute to the pathogenesis of inflammatory or immune-related diseases (Zimmermann and Mcdevitt, 2014). Particularly, MSCs cultivated in 3D structure are sensitive to medium supplemented with HPL, resulting in changes in actin filament formation, mitochondrial dynamics and membrane elasticity that can have an impact on the immunomodulatory function of MSCs (Pasztorek et al., 2019). IL-6 is a regulator of inflammation, and previous reports have shown that IL-6 released from ASCs can increase proliferation of several types of cells (Lu et al., 2016; Pu et al., 2019). Since IL-6 was up-regulated in HPL-cultured ASC sheets, but not in the FBS counterparts, this finding may help to explain the stimulatory effects of conditioned medium of HPL-cultured ASC sheets on the migration and proliferation of fibroblasts *in vitro*. TSG-6 has been identified as an important component of paracrine-mediated MSC immunomodulation (Lee et al., 2014), but the ASC sheets and FBS-cultured monolayer ASCs in the present study exhibited no significant difference in the gene expression of TSG-6. However, FBS or HPL-cultured ASC sheets were found to exhibit significant up-regulation of *CTRP3* relative to monolayer ASCs. CTRP3 was found to inhibit the C-C motif ligand 2 release by macrophages *in vitro* and subsequently reduced the chemotaxis of unstimulated macrophages (Yu et al., 2018). In this study, we confirmed that conditioned medium of HPL-cultured ASC sheets significantly inhibited the migration of macrophages toward LPS-stimulated macrophages *in vitro*, suggesting HPL-cultured ASC sheets can decrease local inflammation by inhibiting macrophage recruitment to the injury site.

Blood flow is required to supply oxygen and nutrition, and to remove waste throughout the process of tissue regeneration. Capillary angiogenesis is therefore vital for promoting regeneration in ischemic tissues. ASCs have been shown to secrete multiple cytokines or growth factors that can promote angiogenesis. Among them, VEGF has been considered to play an important role in ASC-mediated angiogenesis. However, down-regulation of VEGF has been noted in ASCs after sheet formation (Makarevich et al., 2015). After replacing FBS with HPL during the sheet fabrication process, down-regulation of VEGF-A and VEGF-C persisted, but significant up-regulation of HGF was noted. In this study, ELISA also revealed a significantly higher HGF concentration in the conditioned medium of HPL-cultured ASC sheets. HGF production is important for ASC potency by mediating the proangiogenic, prosurvival, and repair promotion activities of ASCs (Cai et al., 2007). A recent clinical trial also demonstrated the effectiveness of topical HGF injection in promoting healing of ischemic ulcers in lower limbs (Gu et al., 2019). Moreover, IL-6 released by ASCs can also stimulate angiogenesis and enhanced recovery after ischemia-reperfusion injury (Pu et al., 2017), suggesting *IL-6* up-regulation in HPL-cultured ASC sheets may also contribute to angiogenesis. Therefore, co-culturing HUVECs with conditioned medium of HPL-cultured ASC sheets revealed more tube-like structure formation of the endothelial cells, indicating the potential of these cell sheets in promoting angiogenesis. By quantification of the capillary area and length in the CAM assay, we confirmed that HPL-cultured ASC sheets significantly promoted angiogenesis, though no significant difference was found between the FBS and HPL-cultured cell sheets.

In summary, we successfully fabricated ASC sheets using HPL as a substitute for xenogeneic FBS, resulting in thicker cell sheets with more abundant ASC-derived ECM deposition. The composition of ECMs within cell sheets was modified by replacing FBS with HPL for ASC culture, affecting the characteristics and function of the resident cells. HPL-cultured ASC sheets more effectively promoted fibroblast migration and proliferation, and they also inhibited the differentiation of fibroblasts into α-SMA-expressing myofibroblasts upon TGF-β1 stimulation *in vitro*. Moreover, FBS and HPL-cultured ASC sheets both inhibited the chemotaxis of unstimulated macrophages, which was possibly mediated through the significant up-regulation of CTRP3. Relative to their FBS counterpart, more HGF was found to release from HPL-cultured ASC sheets, and the angiogenic potential of HPL-cultured ASC sheets was maintained both *in vitro* and *in vivo*. The results of this study demonstrated a promising approach to fabricate ASC sheets with HPL for clinical use. Further animal studies are required to fully extrapolate the cellular function resulting from the altered microenvironment within the HPL-cultured ASC sheets.

## Data Availability Statement

The datasets generated in this study can be found in online repositories. The names of the repository/repositories and accession number(s) can be found in the article/ Supplementary Material.

## Ethics Statement

The study protocol has been approved by the Research Ethics Committee of National Taiwan University Hospital, and the informed consent has been obtained from each donor of adipose tissue.

## Author Contributions

N-CC conceived and designed the experiments, wrote the manuscript, participated in performing the experiments and analyzing the data. Y-KT contributed to the study design, practical work, and manuscript revision. N-HL took part in performing the experiments and analyzing the data. T-HY conceived and designed the experiments and made the final approval of the version to be published. All authors contributed to the article and approved the submitted version.

## Conflict of Interest

The authors declare that the research was conducted in the absence of any commercial or financial relationships that could be construed as a potential conflict of interest.
