# Supplementary figures and images for: Influence of Human Platelet Lysate on Extracellular Matrix Deposition and Cellular Characteristics in Adipose-Derived Stem Cell Sheets
Source: Front Cell Dev Biol. 2020 Oct 22;8:558354. doi: 10.3389/fcell.2020.558354 (PMC7642065; doi:10.3389/fcell.2020.558354)

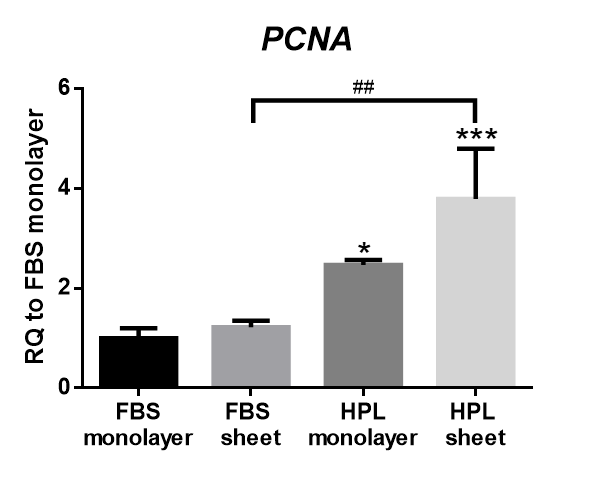

Supplement: Supplementary file 1 [file Image_1.TIF]

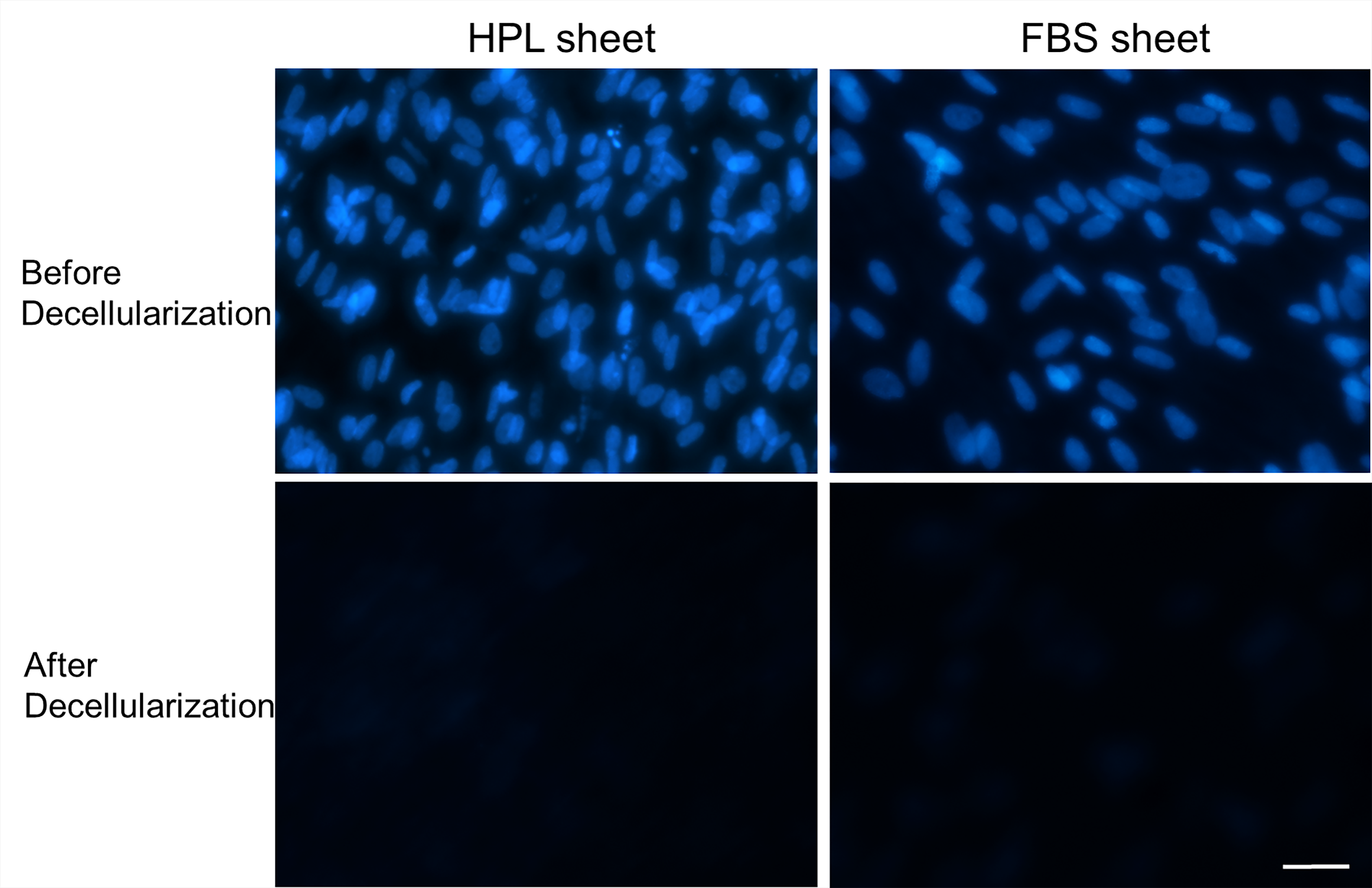

Supplement: Supplementary file 2 [file Image_2.TIF]
