# Supplementary material for: Influence of Human Platelet Lysate on Extracellular Matrix Deposition and Cellular Characteristics in Adipose-Derived Stem Cell Sheets
Source: Front Cell Dev Biol. 2020 Oct 22;8:558354. doi: 10.3389/fcell.2020.558354 (PMC7642065; doi:10.3389/fcell.2020.558354)
Supplement: Supplementary file 3 [file Table_1.DOCX]

**Supplementary Materials**

**Methodology: Proteomic analysis of ASC sheet ECM samples**

1. **Gel Electrophoresis**

Equivalent amount of FBS or HPL-cultured ASC sheet ECM sample was analyzed by 12.5% SDS-PAGE. After electrophoresis, the gels were stained with VisPRO Protein Stain Kit (Visual Protein, Taiwan). After staining, the gels were washed in Milli-Q water and stored at 4 °C until processing for in-gel digestion.

1. **In-Gel Digestion**

The gel lanes corresponding to the sample were cut in 2 slices, and each slice was processed for in-gel digestion according to the method of Shevchenko et al (Shevchenko, A., Tomas, H., Havlis, J., Olsen, J.V., and Mann, M. (2006). In-gel digestion for mass spectrometric characterization of proteins and proteomes. *Nat Protoc* 1**,** 2856-2860. doi: 10.1038/nprot.2006.468). Briefly, slices were washed/dehydrated three times in 50 mM ammonium bicarbonate (pH 7.9) and 50% acetonitrile. Subsequently, cysteine bonds were reduced with 10 mM dithiothreitol for 1 hr at 56°C and alkylated with 25 mM iodoacetamide for 45 min at room temperature in the dark. After two subsequent wash/dehydration cycles, the slices were dried for 10 min in a vacuum centrifuge and incubated overnight with 6.25 ng/μL trypsin in 50 mM ammonium bicarbonate at 25°C. Peptides were extracted once in 100 μL of 1% formic acid and subsequently twice in 100 μL 33of 50% acetonitrile in 5% formic acid. The volume was reduced to 50 μL in a vacuum centrifuge prior to LC-MS/MS analysis.

1. **Nano-LC Separation & Mass Spectrometry.**

Peptides were separated using an Ultimate 3000 nanoLC system (Dionex LC-Packings, Amsterdam, the Netherlands) equipped with a 20 cm × 75 μm i.d. fused silica column custom packed with 3 μm 120 Å ReproSil Pur C18 aqua (Dr. Maisch HPLC GmbH, Ammerbuch-Entringen, Germany). After injection, peptides were trapped at 30 μL/min on a 5 mm × 300 μm i.d. Pepmap C18 cartridge (Dionex LCPackings, Amsterdam, the Netherlands) at 2% buffer B (buffer A, 0.05% formic acid in MQ; buffer B, 80% ACN and 0.05% formic acid in MQ) and separated at 300 nL/min in a 10-40% buffer B gradient in 60 min. Eluting peptides were ionized at 1.7 kV in a Nanomate Triversa Chip-based nanospray source using a Triversa LC coupler (Advion, Ithaca, NJ). Intact peptide mass spectra and fragmentation spectra were acquired on a LTQFT hybrid mass spectrometer (Thermo Fisher Scientific). Intact masses were measured at a resolution of 50 000 in the ICR cell using a target value of 1 × 10^6^ charges. In parallel, following an FT prescan, the top 5 peptide signals (charge-states 2+ and higher) were submitted to MS/MS in the linear ion trap (3 amu isolation width, 30 ms activation, 35% normalized activation energy, Q-value of 0.25 and a threshold of 5000 counts). Dynamic exclusion was applied with a repeat count of 1 and an exclusion time of 30 sec.

1. **Label-free peptide quantification and identification.**

The spectra (Thermo raw files) from tryptic digestion were loaded Proteome discoverer for proteomics software version 2.2 (Thermo Fisher Scientific). The spectra were used to validate identification to MASCOT (www.matrixscience.com) for protein identification. All MS/MS spectra were exported from Proteome discoverer software searched against Homo Sapiens (Human) SwissProt 2018_06 (557,713 sequences; 199,530,821 residues). The search parameters were: peptide mass tolerance set to 20 ppm, MS/MS mass tolerance set at 0.02 Da; up to two missed cleavages were allowed, cysteine carbamidomethylation set as a fixed modification and methionine oxidation set as a variable modification. A decoy database search was performed to determine the peptide false discovery rate (FDR) with the Target Decoy PSM Validator module. A 0.05% peptide FDR threshold was applied. Additionally, all protein identifications had mascot score>25 and protein FDR confidence high level. Then protein quantitation was calculated by unique peptide intensity and normalized by total peptide amount.

1. **System biology analysis.**

The LC-MS/MS data were uploaded from a Microsoft Excel spreadsheet onto FunRich software (http://www.funrich.org) and Ingenuity Pathway Analysis software (https://apps.ingenuity.com). FunRich V3. and IPA 478438M suite generate maps to describe common pathways or molecular connections between control and experiment on the list. Table representations of the molecular relationships between protein were generated using the Core Analysis, based upon processes showing significant association (p<0.05).

**Supplementary Figure 1.** Gene expression of *PCNA* in ASCs under different culture condition (n=3; *p < 0.05, ***p < 0.005 from the control, ##p< 0.01 between the indicated groups).

**Supplementary Figure 2.** DAPI staining revealed complete decellularization of the FBS or HPL-cultured ASC sheets for proteomic analysis (scale bar=200µm).

**Supplementary Table 1.** Primer sequences used for the real-time qPCR analysis

| Target gene |  | Primer sequences |
| --- | --- | --- |
| *COL1A1* | Forward | 5’-CCCTCAAGGTTTCCAAGGAC-3’ |
|  | Reverse | 5’-ACCAGGTTCACCCTTCACAC-3’ |
| *Fibronectin* | Forward | 5’-CTGGCCGAAAATACATTGTAAA-3’ |
|  | Reverse | 5’-CCACAGTCGGGTCAGGAG-3’ |
| Laminin | Forward | 5’-CAGGACCCATTACCCTTTTG-3’ |
|  | Reverse | 5’-GCCCTGCTTGGTTTCTTTATT-3’ |
| *IL-6* | Forward | 5′-CAGTTGCCTTCTCCCTGGG-3′ |
|  | Reverse | 5′-TGAGTGGCTGCTTGTGTGGG-3′ |
| *TSG-6* | Forward | 5’-AAGCACGGTCTGGCAAATACAAGC-3’ |
|  | Reverse | 5’-ATCCATCCAGCAGCACAGACATGA-3’ |
| *CTRP3* | Forward | 5’-CTTTGGAGGCAGCTCATCTATTG-3’ |
|  | Reverse | 5’-CCGGTTTGTGGAGACTCCATG-3’ |
| *IDO-1* | Forward | 5′-CCTGAGGAGCTACCATCTGC-3′ |
|  | Reverse | 5′-TCAGTGCCTCCAGTTCCTTT-3′ |
| *VEGF-A* | Forward | 5′-CTTGCCTTGCTGCTCTACC-3′ |
|  | Reverse | 5′-CACACAGGATGGCTTGAAG-3′ |
| *VEGF-C* | Forward | 5′-CACGAGCTACCTCAGCAAGA-3′ |
|  | Reverse | 5′-GCTGCCTGACACTGTGGTA-3′ |
| *HGF* | Forward | 5’- CTAGATCTTTCCAGTTAATCACACAAC 3’ |
|  | Reverse | 5’- TTCGGAGTCAGTGCCTAAAAGAG-3’ |
| *FGF2* | Forward | 5′-CCGACGGCCGAGTTGAC-3′ |
|  | Reverse | 5′-TGATAGACACAACTCCTCTCTCTTCTG-3′ |
| *PCNA* | Forward | 5’-TTTGGTGCAGCTCACCCTG-3’ |
|  | Reverse | 5’-CGCGTTATCTTCGGCCCTTA-3’ |
| *TGF-β1* | Forward | 5’-CTTCAGCTCCACAGAGAAGAACTG-3’ |
|  | Reverse | 5’-CACGATCATGTTGCACACTGCTCC-3’ |
| *α-SMA* | Forward | 5’-TCTTCCAGCCTTCCTTTA-3’ |
|  | Reverse | 5’-ATGTCAATGTCACACTTCA-3’ |
| *GAPDH* | Forward | 5'-CAAGGCTGAGAACGGGAAGC-3' |
|  | Reverse | 5'-AGGGGGCAGAGATGATGACC-3' |

**Supplementary Table 2.** Protein contents in the FBS and HPL-cultured ASC sheets classified by their relative expression level.
